# Design and in silico validation of polymerase chain reaction primers to detect severe acute respiratory syndrome coronavirus 2 (SARS-CoV-2)

**DOI:** 10.1038/s41598-021-91817-9

**Published:** 2021-06-15

**Authors:** Maria Júlia P. Davi, Selma M. B. Jeronimo, João P. M. S. Lima, Daniel C. F. Lanza

**Affiliations:** 1 Applied Molecular Biology Lab - LAPLIC, Department of Biochemistry, Biosciences Center, Federal University of Rio Grande do Norte, Natal, RN CEP: 59072-970 Brazil; 2 Programa de Pós-Graduação Em Bioinformática (PPg-Bioinfo), Digital Metropolis Institute (IMD), Federal University of Rio Grande do Norte, Natal, RN Brazil; 3 Institute of Tropical Medicine of Rio Grande Do Norte (IMT), Federal University of Rio Grande do Norte, Natal, RN Brazil; 4 Laboratory of Metabolic Systems and Bioinformatics - LASIS, Department of Biochemistry, Biosciences Center, Federal University of Rio Grande do Norte, Natal, RN Brazil; 5 Bioinformatics Multidisciplinary Environment (BioME), Digital Metropolis Institute (IMD), Federal University of Rio Grande do Norte, Natal, RN Brazil

**Keywords:** Diagnostic markers, Pathogens

## Abstract

Accurate designing of polymerase chain reaction (PCR) primers targeting conserved segments in viral genomes is desirable for preventing false-negative results and decreasing the need for standardization across different PCR protocols. In this work, we designed and described a set of primers and probes targeting conserved regions identified from a multiple sequence alignment of 2341 Severe Acute Respiratory Syndrome Coronavirus 2 (SARS-CoV-2) genomes from the Global Initiative on Sharing All Influenza Data (GISAID). We subsequently validated those primers and probes in 211,833 SARS-CoV-2 whole-genome sequences. We obtained nine systems (forward primer + reverse primer + probe) that potentially anneal to highly conserved regions of the virus genome from these analyses. In silico predictions also demonstrated that those primers do not bind to nonspecific targets for human, bacterial, fungal, apicomplexan, and other Betacoronaviruses and less pathogenic sub-strains of coronavirus. The availability of these primer and probe sequences will make it possible to validate more efficient protocols for identifying SARS-CoV-2.

## Introduction

Identifying viral genetic material using the PCR technique is considered the gold standard for determining SARS-CoV-2 in nasal swab samples from symptomatic patients. Since the outbreak started, the World Health Organization (WHO) released some SARS-CoV-2 polymerase chain reaction (PCR) protocol assays produced by different reference institutions in the world^1^. In addition to these initial protocols, an increasing number of works and commercial kits suggest new alternatives to identifying SARS-CoV-2 and its recent variants by molecular or immunological approaches^2–4^.

Concomitant with those advances, the rapid increase in the number of available SARS-CoV-2 sequences from different localities identified several polymorphic regions in SARS-CoV-2's genome. Therefore, it is plausible that some of the available early PCR detection kits have primers that may target these polymorphic regions. That possibility can compromise accurate identification of some viral variants and increase the number of false negatives or inconclusive results, especially with the rise of new and potentially dangerous variants.

In this context, the development of primers that target conserved regions in the genome to detect many viral variants is imperative. In this work, we identified 26 conserved segments (CS) in the SARS-CoV-2 genome based on an alignment of 2,341 full genome sequences and used these regions as a target for the design of universal primers and probes. We extended the analyses to include 211,833 SARS-CoV-2 sequences and the recent virus variants and further demonstrated that the proposed primers are still located in conserved regions, confirming their potential as universal primers.

## Results

At the end of the analysis, we elected nine candidate systems (forward primer + reverse primer + probe) that met all requirements (Table 1). In general, in silico analyses revealed that the primers pairs proposed in this study (UFRN_primers) (Table 1) are more compatible with each other (evaluated by lower differences between forward and reverse primers' Tm), have lower self-complementarity (both overall and 3'), and higher specificity than the previously described primers (PD_primers) (Table 2). Regarding the proposed probes, only the probes UFRN_3_P and UFRN_4_P did not reach a Tm higher than that of their respective primer pairs.

**Table 1:** Primers designed in this study.

Primer name	Sequence 5'→ 3'	Length	Tm	GC (%)	SC	SC 3'	Target	Size	No mis	10% of mis
UFRN_1_F	GGGCATACACTCGCTATGTC	20	58.22	55	4	3	ORF1a	101	209,537 (98.91%)	211,355 (99.77%)
UFRN_1_R	GCATGAAGCTTTACCAGCAC	20	57.73	50	6	0				
UFRN_1_P	TCTGTGGCCCTGATGGCTACCCT	23	67.22	60.87	7	2				
UFRN_2_F	GGCTACTAACAATGCCATGC	20	57.22	50	5	2	ORF1a	137	208,356 (98.35%)	209,352 (98.82%)
UFRN_2_R	TAACATTTGGGCCGACAACA	20	58.02	45	4	1				
UFRN_2_P	GGGTGGTAGTTGTGTTTTAAGCGG	24	62.33	50	4	1				
UFRN_3_F	TTCATGTTGTCGGCCCAAAT	20	58.37	45	4	3	ORF1a	98	207,689 (98.04%)	209,036 (98.67%)
UFRN_3_R	TGGTGCAAGTAGAACTTCGT	20	57.1	45	5	3				
UFRN_3_P	GAAGACATTCAACTTCTTAAGAGTGC	26	58.71	38.46	8	4				
UFRN_4_F	TGGTGCTAGGAGAGTGTGG	19	58.33	57.89	4	0	ORF1a	95	209,233 (98.77%)	210,535 (99.38%)
UFRN_4_R	CCCACATGGAAATGGCTTGAT	21	58.89	47.62	4	2				
UFRN_4_P	CTTATGAATGTCTTGACACTCGTTTATA	28	58.01	32.14	8	4				
UFRN_5_F	AGGGCACACTAGAACCAGAA	20	58.27	50	4	0	ORF1b	105	209,501 (98.89%)	210,753 (99.49%)
UFRN_5_R	CAATTTCAGCAGGACAACGC	20	58.31	50	4	2				
UFRN_5_P	GGTCCAGACATGTTCCTCGGAACT	24	64.18	54.17	8	6				
UFRN_6_F	TCTTCACGACATTGGTAACCC	21	57.95	47.62	5	3	ORF1b	90	210,278 (99.26%)	210,890 (99.55%)
UFRN_6_R	TCACTACAAGGCTGTGCATC	20	57.9	50	4	2				
UFRN_6_P	TACCTCAAGCTGATGTAGAATGGAAG	26	60.41	42.31	8	0				
UFRN_7_F	CTTCACGACATTGGTAACCCT	21	57.95	47.62	5	1	ORF1b	90	210,240 (99.24%)	210,884 (99.55%)
UFRN_7_R	GTCACTACAAGGCTGTGCAT	20	58.19	50	4	2				
UFRN_7_P	GTGTACCTCAAGCTGATGTAGAATGG	26	61.4	46.15	8	0				
UFRN_8_F	GGCACAGGTGTTCTTACTGA	20	57.46	50	4	1	S	107	210,860 (99.54%)	211,488 (99.83%)
UFRN_8_R	TCAAGTGTCTGTGGATCACG	20	57.56	50	4	2				
UFRN_8_P	CCAACAATTTGGCAGAGACATTGC	24	61.62	45.83	5	3				
UFRN_9_F	AGGCACAGGTGTTCTTACTG	20	57.45	50	4	1	S	93	210,309 (99.28%)	211,491 (99.83%)
UFRN_9_R	TCACGGACAGCATCAGTAGT	20	58.45	50	3	2				
UFRN_9_P	TCCAACAATTTGGCAGAGACATTGC	25	62.75	44	5	3				

The percentage of the total number of sequences that annel without mismatches or allowing 10% mismatches are shown in parentheses.

*F* forward primer; *R* reverse primer; *P* probe; *Tm* melting temperature; GC% = G + C percentage; *SC* self complementarity; *SC 3’* self 3’-complementarity; *No mis* number of sequences that anneal to the primer without mismatches; *10% mis* number of sequences that anneal to the primer allowing 10% mismatches.

**Table 2:** Primers released by WHO to detect SARS-CoV-2 using polymerase chain reaction.

Location/primer or probe name	Sequence (5′ > 3′)	Length	Tm	GC (%)	SC	SC 3'	Target	Size	Specificity	
									No mis	10% mis
**Germany (17 January 2020)**										
RdRP_SARSr-F2	GTGARATGGTCATGTGTGGCGG	22	63.25	57.14	5.5	1	RdRp	100	4 (0.001%)	210,600 (99.41%)
RdRP_SARSr-R1	CARATGTTAAASACACTATTAGCATA	26	54.25	25	5	4	RdRp			
RdRP_SARSr-P2	FAM-CAGGTGGAACCTCATCAGGAGATGC-BBQ	25	64.89	56	6	5	RdRp			
E_Sarbeco_F1	ACAGGTACGTTAATAGTTAATAGCGT	26	58.29	34.62	8	8	E	113	210,071 (99.16%)	210,610 (99.42%)
E_Sarbeco_R2	ATATTGCAGCAGTACGCACACA	22	60.93	45.45	7	1	E			
E_Sarbeco_P1	FAM-ACACTAGCCATCCTTACTGCGCTTCG-BBQ	26	66.78	53.85	4	2	E			
N_Sarbeco_F1	CACATTGGCACCCGCAATC	19	60.15	57.89	4	0	N	128	205,051 (96.79%)	209,594 (98.94%)
N_Sarbeco_R1	GAGGAACGAGAAGAGGCTTG	20	58	55	3	1	N			
N_Sarbeco_P1	AM-ACTTCCTCAAGGAACAACATTGCCA-BBQ	25	63.15	44	8	3	N			
**Hong Kong (23 January 2020)**										
HKU-ORF1b-nsp14F	TGGGGYTTTACRGGTAACCT	20	47.07	50	7.5	4.5	ORF1b	132	209,633 (98.96%)	211,205 (99.70%)
HKU- ORF1b-nsp14R	AACRCGCTTAACAAAGCACTC	21	53.44	45	4	0	ORF1b			
HKU-ORF1b-nsp141P	FAM-TAGTTGTGATGCWATCATGACTAG-TAMRA	24	54.86	39.13	10.5	6,5	ORF1b			
HKU-NF	TAATCAGACAAGGAACTGATTA	22	52.27	31.82	7	7	N	110	207,359 (97.88%)	209,580 (98.93%)
HKU-NR	CGAAGGTGTGACTTCCATG	19	55.95	52.63	4	4	N			
HKU-NP	FAM-GCAAATTGTGCAATTTGCGG-TAMRA	20	58.05	45	14	6	N			
**China (24 January 2020)**										
ORF1ab_F	CCCTGTGGGTTTTACACTTAA	21	55.7	42.86	4	4	ORF1ab	119	205,630 (97.07%)	206,591 (97.52%)
ORF1ab_R	ACGATTGTGCATCAGCTGA	19	57.46	47.37	8	8	ORF1ab			
ORF1ab_P	FAM-CCGTCTGCGGTATGTGGAAAGGTTATGG-BHQ1	28	67.24	53.57	3	0	ORF1ab			
N_F	GGGGAACTTCTCCTGCTAGAAT	22	59.23	50	7	2	N	99	124,314 (58.68%)	131,849 (62.24%)
N_R	CAGACATTTTGCTCTCAAGCTG	22	58.18	45.45	4	2	N			
N_P	FAM-TTGCTGCTGCTTGACAGATT-TAMRA	20	58.39	45	4	1	N			
**Japan (24 January 2020)**										
NIID_WH-1_F501-F	TTCGGATGCTCGAACTGCACC	21	63.27	57.14	4	0	ORF1a	413	208,053 (98.21%)	210,374 (99.31%)
NIID_WH-1_R913-R	CTTTACCAGCACGTGCTAGAAGG	23	61.47	52.17	10	10	ORF1a			
NIID_WH-1_F509-F	CTCGAACTGCACCTCATGG	19	58.24	57.89	4	2	ORF1a	346	206,148 (97.31%)	210,255 (99.25%)
NIID_WH-1_R854-R	CAGAAGTTGTTATCGACATAGC	22	55.05	40.91	4	3	ORF1a			
NIID_WH-1_Seq_F519	ACCTCATGGTCATGTTATGG	20	54.79	45	6	1	ORF1a	322	206,749 (97.59%)	208,982 (98.65%)
NIID_WH-1_Seq_R840	GACATAGCGAGTGTATGCC	19	55.61	52.63	4	3	ORF1a			
WuhanCoV-spk1-f	TTGGCAAAATTCAAGACTCACTTT	24	58.02	33.33	5	3	S	547	207,888 (98.13%)	209,802 (99.04%)
WuhanCoV-spk2-r	TGTGGTTCATAAAAATTCCTTTGTG	25	56.98	32	4	3	S			
NIID_WH-1_F24381	TCAAGACTCACTTTCTTCCAC	21	55.48	42.86	4	0	S	493	207,271 (98.74%)	209,820 (99.04%)
NIID_WH-1_R24873	ATTTGAAACAAAGACACCTTCAC	23	56.13	34.78	5	0	S			
NIID_WH-1_Seq_F24383	AAGACTCACTTTCTTCCACAG	21	55.47	42.86	4	1	S	483	207,222 (97.82%)	209,803 (99.04%)
NIID_WH-1_Seq_R24865	CAAAGACACCTTCACGAGG	19	55.88	52.63	3	2	S			
NIID_2019-nCOV_N_F2	AAATTTTGGGGACCAGGAAC	20	56.09	45	6	1	N	108	0 (0%)	209,526 (98.91%)
NIID_2019-nCOV_N_R2	TGGCAGCTGTGTAGGTCAAC	20	60.25	55	6	2	N			
NIID_2019-nCOV_N_P2	FAM-ATGTCGCGCATTGGCATGGA-BHQ	20	63.5	55	6	0	N			
**Thailand (23 January 2020)**										
WH-NIC N-F	CGTTTGGTGGACCCTCAGAT	20	59.68	55	4	2	N	57	207,825 (98.10%)	211,075 (99.64%)
WH-NIC N-R	CCCCACTGCGTTCTCCATT	19	60	57.89	3	1	N			
WH-NIC N-P	FAM-CAACTGGCAGTAACCA- BQH1	16	50.27	50	7	1	N			
**USA (24 January 2020)**										
2019-nCoV_N1-F	GACCCCAAAATCAGCGAAAT	20	56.67	45	2	2	N	72	208,464 (98.40%)	211,123 (99.66%)
2019-nCoV_N1-R	TCTGGTTACTGCCAGTTGAATCTG	24	60.8	45.83	7	5	N			
2019-nCoV_N1-P	FAM-ACCCCGCATTACGTTTGGTGGACC-BHQ1	24	67.48	58.33	4	4	N			
2019-nCoV_N2-F	TTACAAACATTGGCCGCAAA	20	57.11	40	5	5	N	67	204,237 (96.41%)	209,575 (98.93%)
2019-nCoV_N2-R	GCGCGACATTCCGAAGAA	18	58.53	55.56	5	2	N			
2019-nCoV_N2-P	FAM-ACAATTTGCCCCCAGCGCTTCAG-BHQ1	23	66.45	56.52	6	2	N			
2019-nCoV_N3-F	GGGAGCCTTGAATACACCAAAA	22	58.84	45.45	4	0	N	72	208,807 (98.57%)	210,931 (99.57%)
2019-nCoV_N3-R	TGTAGCACGATTGCAGCATTG	21	59.87	47.62	5	3	N			
2019-nCoV_N3-P	FAM-AYCACATTGGCACCCGCAATCCTG-BHQ1	24	65.21	56.52	4	1	N			
RP-F	AGATTTGGACCTGCGAGCG	19	60.45	57.89	3	2	RNAse P	0	0 (0%)	0 (0%)
RP-R	GAGCGGCTGTCTCCACAAGT	20	62.44	60	5	2	RNAse P			
RP-P	FAM-TTCTGACCTGAAGGCTCTGCGCG-BHQ1	23	67.21	60.87	4	4	RNAse P			
**Paris (2 March 2020)**										
nCoV_IP2-12669Fw	ATGAGCTTAGTCCTGTTG	18	51.11	44.44	4	0	RdRp	108	209,365 (98.83%)	210,516 (99.37%)
nCoV_IP2-12759Rv	CTCCCTTTGTTGTGTTGT	18	52.57	44.44	1	0	RdRp			
nCoV_IP2-12696bProbe( +)	HEX-AGATGTCTTGTGCTGCCGGTA-BHQ-1	21	61.78	52.38	4	4	RdRp			
nCoV_IP4-14059Fw	GGTAACTGGTATGATTTCG	19	50.65	42.11	3	2	RdRp	107	210,158 (99.20%)	211,239 (99.71%)
nCoV_IP4-14146Rv	CTGGTCAAGGTTAATATAGG	20	49.98	40	4	0	RdRp			
nCoV_IP4-14084Probe( +)	FAM-TCATACAAACCACGCCAGG-BHQ-1	19	57.76	52.63	3	3	RdRp			
E_Sarbeco_F1	ACAGGTACGTTAATAGTTAATAGCGT	26	58.29	34.62	8	8	E	113	210,071 (99.16%)	210,610 (99.42%)
E_Sarbeco_R2	ATATTGCAGCAGTACGCACACA	22	60.93	45.45	7	1	E			
E_Sarbeco_P1	FAM-ACACTAGCCATCCTTACTGCGCTTCG-BHQ-1	26	66.78	53.85	4	2	E			

The percentage of the total number of sequences that annel without mismatches or allowing 10% mismatches are shown in parentheses.

*F* forward primer; *R* reverse primer; *P* probe; *Tm* melting temperature; *GC%* G + C percentage; *SC* self complementarity; *SC 3*’ self 3’-complementarity; *No mis* number of sequences that anneal to the primer without mismatches; *10% mis* number of sequences that anneal to the primer allowing 10% mismatches.

By comparing the number of SARS-CoV-2 sequences that anneal without mismatches ("No mis" in Tables 1 and 2), using in silico PCR methodologies, it is safe to assume that the set of UFRN_primers targets fewer polymorphic sites in the viral genome than the PD_primers set. Among the 211,833 SARS-CoV-2 genomic sequences used as targets, UFRN_primers anneal with 100% identity with at least 207,689 (UFRN_3) reaching 210,860 (UFRN_8). The probes from the UFRN_primers set aligned with most templates in BLAST searches against the same sequence database (Supplementary material 1 and 2).

We also compared the UFRN_primers against a specific sequence set containing recent SARS-CoV-2 variants, which includes (B.1.1.7, B.1.351, B.1.427, B.1.429, B.1.525, and P.1) (Table 3). At this test, the proposed primer set also presented better in silico results when compared to the PD_primers set. All UFRN_primers, except for the UFRN_4 primer, annealed with all the sequences from variants B.1351 (495 sequences), B.1429, B.1427, B.1525 (94 sequences in total), and P1 (177 sequences) tested, with no mismatches allowed. Regarding the B.1.1.7 variant, the primers UFRN_3, UFRN_5, and UFRN_8 annealed to the vast majority of its sequences (Table 3). Still, two primers (2019-nCoV_N2 and nCoV_IP2-12669Fw) from the PD_primer set had the same performance as the three UFRN_primers mentioned above (Table 4).

**Table 3:** Analysis of potential annealing (In silico PCR) of UFRN primers (UFRN_primers) to the genomes of the main SARS-CoV-2 variants.

Primer name	SARS-CoV-2 Variant	No mismatches	10% mismatches
UFRN_1	B.1.1.7	1906 (98.70%)	1931 (100%)
	B.1351	495 (100%)	495 (100%)
	P.1	177 (100%)	177 (100%)
	B.1429 + 1427 + 1525	94 (100%)	94 (100%)
UFRN_2	B.1.1.7	1918 (99.32%)	1931 (100%)
	B.1351	495 (100%)	495 (100%)
	P.1	177 (100%)	177 (100%)
	B.1429 + 1427 + 1525	94 (100%)	94 (100%)
UFRN_3	B.1.1.7	1930 (99.94%)	1931 (100%)
	B.1351	495 (100%)	495 (100%)
	P.1	177 (100%)	177 (100%)
	B.1429 + 1427 + 1525	94 (100%)	94 (100%)
UFRN_4	B.1.1.7	1915 (99.17%)	1930 (99.94%)
	B.1351	487 (98.38%)	495 (100%)
	P.1	177 (100%)	177 (100%)
	B.1429 + 1427 + 1525	92 (97.87%)	94 (100%)
UFRN_5	B.1.1.7	1931 (100%)	1931 (100%)
	B.1351	495 (100%)	495 (100%)
	P.1	177 (100%)	177 (100%)
	B.1429 + 1427 + 1525	94 (100%)	94 (100%)
UFRN_6	B.1.1.7	1928 (99.84%)	1931 (100%)
	B.1351	495 (100%)	495 (100%)
	P.1	177 (100%)	177 (100%)
	B.1429 + 1427 + 1525	93 (98.93%)	94 (100%)
UFRN_7	B.1.1.7	1926 (99.74%)	1931 (100%)
	B.1351	495 (100%)	495 (100%)
	P.1	177 (100%)	177 (100%)
	B.1429 + 1427 + 1525	93(98.93%)	94 (100%)
UFRN_8	B.1.1.7	1930 (99.94%)	1930 (99.94%)
	B.1351	495 (100%)	495 (100%)
	P.1	177 (100%)	177 (100%)
	B.1429 + 1427 + 1525	94 (100%)	94 (100%)
UFRN_9	B.1.1.7	1928 (99.84%)	1931 (100%)
	B.1351	495 (100%)	495 (100%)
	P.1	177 (100%)	177 (100%)
	B.1429 + 1427 + 1525	94 (100%)	94 (100%)

The percentage of the total number of sequences that annel without mismatches or allowing 10% mismatches are shown in parentheses.

*No mis* number of sequences that anneal to the primer without mismatches; *10% mis* number of sequences that anneal to the primer allowing 10% mismatches.

**Table 4:** Analysis of potential annealing (In silico PCR) of WHO primers (PD_primers) to the genomes of the main SARS-CoV-2 variants.

Location/ primer name	Sars-CoV-2 variant	No mismatches	10% mismatches
**Germany (17 January 2020)**			
RdRp	B.1.1.7	0(0%)	1931 (100%)
	B.1351	0(0%)	495 (100%)
	P.1	0(0%)	177 (100%)
	B.1429 + 1427 + 1525	0(0%)	94 (100%)
E_Sarbeco	B.1.1.7	1926 (99.74%)	1931 (100%)
	B.1351	495 (100%)	495 (100%)
	P.1	177 (100%)	177 (100%)
	B.1429 + 1427 + 1525	94 (100%)	94 (100%)
N_Sarbeco	B.1.1.7	1924 (99.63%)	1930 (99.94%)
	B.1351	494 (99.79%)	495 (100%)
	P.1	177 (100%)	177 (100%)
	B.1429 + 1427 + 1525	94 (100%)	94 (100%)
**Hong Kong (23 January 2020)**			
HKU-ORF1b-nsp14	B.1.1.7	1926 (99.74%)	1930 (99.94%)
	B.1351	495 (100%)	495 (100%)
	P.1	177 (100%)	177 (100%)
	B.1429 + 1427 + 1525	93 (99.93%)	94 (100%)
HKU-N	B.1.1.7	1923 (99.58%)	1931 (100%)
	B.1351	493 (99.59%)	494 (99.79%)
	P.1	177 (100%)	177 (100%)
	B.1429 + 1427 + 1525	94 (100%)	94 (100%)
**China (24 January 2020)**			
ORF1ab	B.1.1.7	1927 (99.79%)	1929 (99.89%)
	B.1351	495 (100%)	495 (100%)
	P.1	177 (100%)	177 (100%)
	B.1429 + 1427 + 1525	93 (99.93%)	94 (100%)
N	B.1.1.7	3 (0.15%)	3 (0.15%)
	B.1351	0(0%)	494 (99.79%)
	P.1	0(0%)	0(0%)
	B.1429 + 1427 + 1525	0(0%)	94 (100%)
**Japan (24 January 2020)**			
NIID_WH-1_F501	B.1.1.7	1925 (99.68%)	1931 (100%)
	B.1351	495 (100%)	495 (100%)
	P.1	177 (100%)	177 (100%)
	B.1429 + 1427 + 1525	94 (100%)	94 (100%)
NIID_WH-1_F509	B.1.1.7	1912 (99.01%)	1931 (100%)
	B.1351	491 (99.19%)	494 (99.79%)
	P.1	177 (100%)	177 (100%)
	B.1429 + 1427 + 1525	94 (100%)	94 (100%)
NIID_WH-1_Seq_F519	B.1.1.7	1909 (98.86%)	1928 (99.84%)
	B.1351	490 (98.98%)	491 (99.19%)
	P.1	177 (100%)	177 (100%)
	B.1429 + 1427 + 1525	93 (99.93%)	94 (100%)
WuhanCoV-spk1	B.1.1.7	1926 (99.74%)	1931 (100%)
	B.1351	493 (99.59%)	495 (100%)
	P.1	177 (100%)	177 (100%)
	B.1429 + 1427 + 1525	89 (94.68%)	94 (100%)
NIID_WH-1_F24381	B.1.1.7	1917 (99.27%)	1931 (100%)
	B.1351	494 (99.79%)	495 (100%)
	P.1	177 (100%)	177 (100%)
	B.1429 + 1427 + 1525	88 (93.61%)	94 (100%)
NIID_WH-1_Seq_F24383	B.1.1.7	1915 (99.17%)	1931 (100%)
	B.1351	494 (99.79%)	495 (100%)
	P.1	177 (100%)	177 (100%)
	B.1429 + 1427 + 1525	88 (93.61%)	94 (100%)
NIID_2019-nCOV_N_	B.1.1.7	0(0%)	1931 (100%)
	B.1351	0(0%)	494 (99.79%)
	P.1	0(0%)	177 (100%)
	B.1429 + 1427 + 1525	0(0%)	94 (100%)
**Thailand (23 January 2020)**			
WH-NIC_N	B.1.1.7	1921 (99.48%)	1931 (100%)
	B.1351	492 (99.39%)	495 (100%)
	P.1	177 (100%)	177 (100%)
	B.1429 + 1427 + 1525	93 (99.93%)	94 (100%)
**USA (24 January 2020)**			
2019-nCoV_N1	B.1.1.7	1919 (99.37%)	1931 (100%)
	B.1351	476 (96.16%)	495 (100%)
	P.1	177 (100%)	177 (100%)
	B.1429 + 1427 + 1525	92 (97.87%)	94 (100%)
2019-nCoV_N2	B.1.1.7	1930 (99.94%)	1931 (100%)
	B.1351	486 (98.18%)	494 (99.79%)
	P.1	177 (100%)	177 (100%)
	B.1429 + 1427 + 1525	93 (99.93%)	94 (100%)
2019-nCoV_N3	B.1.1.7	1817 (94.09%)	1931 (100%)
	B.1351	494 (99.79%)	495 (100%)
	P.1	174 (98.30%)	177 (100%)
	B.1429 + 1427 + 1525	92 (97.87%)	94 (100%)
RP	B.1.1.7	0(0%)	0(0%)
	B.1351	0(0%)	0(0%)
	P.1	0(0%)	0(0%)
	B.1429 + 1427 + 1525	0(0%)	0(0%)
**Paris (2 March 2020)**			
nCoV_IP2-12669Fw	B.1.1.7	1930 (99.94%)	1931 (100%)
	B.1351	493 (99.59%	495 (100%)
	P.1	176 (99.43%)	177 (100%)
	B.1429 + 1427 + 1525	93 (99.93%)	94 (100%)
nCoV_IP4-14059Fw	B.1.1.7	1924 (99.63%)	1931 (100%)
	B.1351	495 (100%)	495 (100%)
	P.1	177 (100%)	177 (100%)
	B.1429 + 1427 + 1525	93 (99.93%)	94 (100%)
E_Sarbeco	B.1.1.7	1926 (99.74%)	1931 (100%)
	B.1351	495 (100%)	495 (100%)
	P.1	177 (100%)	177 (100%)
	B.1429 + 1427 + 1525	94 (100%)	94 (100%)

The percentage of the total number of sequences that annel without mismatches or allowing 10% mismatches are shown in parentheses.

*No mis* number of sequences that anneal to the primer without mismatches; *10% mis* number of sequences that anneal to the primer allowing 10% mismatches.

Concerning the specificity, both primers set performed well. Tests allowing 20% mismatch against Apicomplexa targets revealed that the 2019-nCoV_N2-F / 2019-nCoV_N2-R and UFRN_8_F / UFRN_8_R primer pairs could generate 746 bp and 755 bp amplicons with *Toxoplasma gondii* sequences from accession codes XTG08368.2 and XM_002364674.2, respectively. The other pairs of primers did not present nonspecific amplicons allowing values between 0 and 20% of mismatches.

Examining the genomes of Gammacoronavirus, Alphacoronavirus, SARS-CoV, SARS-CoV-like, MERS-CoV, Betacoronavirus (excluding SARS-CoV-2), only two (UFRN_1 and UFRN_5) of the nine primers produced amplicons, only with mismatches' allowance (10%). Against 2298 sequences retrieved from the Virus Variation database, these tests predicted just one amplicon (101 bp) with one sequence target (accession code MG772934.1) and 137 amplicons (105 bp) from primer UFRN_1 and UFRN_5, respectively.

## Discussion

Early detection of pathogens is crucial to disease prevention^5^ and containment, especially during epidemic outbreaks^6^. PCR is a reliable and relatively accessible molecular method that directly recognizes pathogen-derived material from patients samples^7^. However, PCR protocols' optimization is strongly dependent on primers' specificity and efficiency^8^. This reason, combined with the increasing number of SARS-CoV-2 sequences available and its crescent polymorphism, led us to design a set of new primers that can address very conserved regions of the virus genomes.

Therefore, to aid PCR optimization, the UFRN_primers were designed to present Tm values that were as close as possible. These settings will probably enable the use of at least two systems using the same thermal cycling parameters. In this way, it would be possible to perform the PCR test identifying different viral genome regions simultaneously, according to the protocols already described for the PD_primers. In this context, possibly the systems UFRN_3 and UFRN_4 will have different thermal cycling parameters compared to the other systems since, in this case, the probe Tm is similar to the primers (Table 1). Probably these systems will depend on more annealing time to ensure that the probe has interacted in the DNA template before the amplification starts.

The higher specificity of UFRN_primers confirmed by in silico analysis is mainly due to the availability of 2.341 genome sequences, which made it possible to identify the conserved regions with greater accuracy from the alignment. The UFRN_6 and UFRN_7 primers differ only by one base and have overlapping probes. However, these discrete differences were sufficient to alter the sequences in which these primers interact (Table 1). Only 12 sequences did not anneal with the designed primers. Among them, seven were isolated from pangolins and one from bats, all from China provinces. The other four sequences are from Australia and Nigeria and presented a high percentage of N bases, which might have caused negative results.

Another striking result is that UFRN_primers presented a higher potential to identify the main SARS-CoV-2 recent variants of concern than the PD_primers, significantly the B.1.351, B.1.427, B.1.429, B.1.525, and P.1. In silico predictions indicate that the UFRN_primers are potentially less prone to generate false-negative results. Its application could represent a significant difference to Covid-19 diagnostic and epidemiology since the Food and Drugs Administration (FDA) has recently warned of the negative impact of SARS-CoV-2 genetic variants on molecular detection tests available^9^.

The use of universal primers makes it possible to identify several virus variants using the same PCR protocol. UFRN_primers are strong candidates to simplify the procedures and supply chain for detecting SARS-CoV-2, allowing, for example, the mass production of primers and kits that could be applied in different parts of the world with equivalent efficiency. However, the primers presented here still depend on in vitro validation. The availability of these sequences at this time will be crucial so that these new protocols can be validated promptly to assist in the control of the SARS-CoV-2 pandemic.

Another critical point is that primers presented here were tested against the updated RNA sequences databases from bacteria, fungi, and protozoa and did not generate nonspecific amplicons in any case. Although executed through in silico analyses, this lack of prediction increases the potential for applying these primers to different samples such as blood, feces, or even environmental samples. Currently, the most suitable sample for detecting SARS-CoV-2 is the human nasal swab; however, there are already studies that have shown digestive symptoms (e.g. diarrhea and vomiting)^10,11^ and other less frequent symptoms (e.g. conjunctivitis) in patients who tested positive for SARS-CoV-2^12–14^. This diversity of symptoms makes clinical diagnosis difficult, and testing new types of samples may be needed quickly. The application of UFRN_primers to detect SARS-CoV-2 in blood or fecal samples is likely efficient since these primers should not interact non-specifically with RNAs of the main protozoa and bacteria that cause health problems in humans.

Quite possibly, at the time of publication of this work, a considerably larger number of additional sequences will be available, which may reveal new polymorphic sites in the target regions of UFRN_primers and PD_primers. In this way, our research group will continue this bioinformatics work, and whenever relevant, we will report new updates on the primer sequences or new primers.

## Methods

Whole-genome sequences of SARS-CoV-2 from human isolates were retrieved from the Global Initiative on Sharing All Influenza Data (GISAID—gisaid.org)^15^ and Virus Variation from the National Center for Biotechnology Information (NCBI—https://www.ncbi.nlm.nih.gov/genome/viruses/variation/)^16^ databases, between Mar 30 and Nov 24, 2020. To minimize sequencing errors and artifacts, we activated the filters "*complete (*> *29.000 bp)*", "*high coverage only*" and "*low coverage excl*" at sequence retrieval in GISAID database and the filter "*Complete*" under the option "*Nucleotide completeness*" from the Virus Variation database. The full list of authors and laboratories of GISAID submissions and the Virus Variation sequences accessions are available in Supplementary Table 3.

Complete fasta sequences were then aligned using Clustal-Omega, version 1.2.4^17^, with standard parameters, using a supercomputer. To avoid excessive misaligned gaps and to better identify conserved polymorphic sites, we trimmed the multiple sequence alignments (MSAs) using the trimAL tool, version 1.2^18^, with the *"-automated1*" option. We used the sequence from a Wuhan seafood market pneumonia virus (GenBank Accession code MN908947)^19^ as a reference for all alignments to identify site and region positions.

The CSs were submitted to online Primer-BLAST^20^ to design primer pairs adopting the following criteria: PCR product size = 90–150 nt; primer melting temperatures (°C) minimum = 55, optimum = 58, maximum = 63 and maximum melting temperature (Tm) difference = 2 °C. The specificity check was performed using the complete Refseq RNA databases for *Homo sapiens* (taxid: 9606), Bacteria (taxid: 2), Fungi (taxid:4751), Apicomplexa (taxid:5794). We set the primer specificity stringency so that the primer must have at least 3 total mismatches to unintended targets, including at least 2 mismatches within the last 5 bps at the 3' ignoring targets with 5 or more mismatches to the primer. The other Primer-BLAST parameters have been kept in the default configuration to confirm the newly-designed primers pairs features.

From all the primers generated by the Primer-BLAST, we selected 124 primer pairs that presented low self-complementarity for total annealing (max 5 nt) and also for annealing in the 3' region (max 3 nt). After individual evaluation using the Geneious suite (version 9.1.8, 2017), we elected 9 primer pairs that target regions with 100% identity among all 2143 initial genomes. These primers comprise ORF1a, ORF1b, and S regions of the SARS-CoV-2 genome. TaqMan probes for each primer pair were also designed considering the same alignment and prioritizing conserved regions inside each of the predicted amplicons.

To compare and assess the already used and newly-designed primers and probes' annealing specificity, we used three different tools: PrimerSearch version 6.6.0 from the Emboss package^21^, the stand-alone BLAST + ^22^, and the on-line Primer-BLAST. For the first two tools, we used five different custom databases: (1) SARS-CoV-2 sequences from GISAID (211,833 genome sequences retrieved on Nov 24, 2020), with the filters as mentioned earlier activated; (2) SARS-CoV-2 sequences from Virus Variation; (3) RefSeq RNAs from Apicomplexa taxon, retrieved from GenBank on Mar 30, 2020; (4) RefSeq RNAs from Toxoplasma taxon, also from GenBank (Mar 30, 2020) and (5) 2298 sequences from Virus Variation database, including Gammacoronavirus, Alphacoronavirus, SARS-CoV, SARS-CoV-like, MERS-CoV, Betacoronavirus (excluding Sars-CoV-2).

The specificity test's first step was to search all 5' and 3' primers pairs sequences to verify amplicon possibilities using PrimerSearch, against each of the databases mentioned above. We used three different mismatch allowance percentages (0, 10, and 20%). We also evaluated the number of hits subject sequences from stand-alone BLAST +, the aligned start and end regions, and the number of mismatches for each alignment for probes similarity searches.

The genome sequences of B.1.1.7, B.1.351, B.1.427, B.1.429, B.1.525, and P.1 variants were retrieved from the GISAID database with the following filters activated: "complete sequence", "excl low coverage", "high coverage", and "w/ pacient status". The total number of sequences for each variant was: 1931 for B.1.1.7, 495 for B.1.351, 94 for B.1.427, B.1.429 e B.1.525, and 177 for P.1. The primer pairs were aligned with each set of sequences using PrimerSearch, with the parameters of 0% mismatches and 10% mismatches allowed. The results were processed and recorded for each primer pair and variant using a custom shell script.

## Supplementary Information

Supplementary Information 1.

Supplementary Information 2.

## Data Availability

The sequences utilized during the current study are publicly available in GISAID (https://www.gisaid.org/) and Virus Variation (https://www.ncbi.nlm.nih.gov/genome/viruses/variation/) databases. Sequence codes are available in Supplementary Material 3. Any other data/protocol is open upon request to the corresponding author.

## References

[1] WHO. https://www.who.int/emergencies/diseases/novel-coronavirus-2019/technical-guidance/laboratory-guidance (2020).

[2] Chan JF (2020). Improved molecular diagnosis of COVID-19 by on the novel, highly sensitive and specific COVID-19-RdRp/Hel real-time reverse transcription-polymerase chain reaction assay validated in vitro and with clinical specimens. J. Clin. Microbiol..

[3] Wang, Y., Kang, H., Liu, X. & Tong, Z. Y. Combination of RT qPCR Testing and clinical features for diagnosis of COVID-19 facilitates management of SARS-CoV-2 outbreak. *J. Med. Virol*. 10.1002/jmv.25721 (2020). 10.1002/jmv.25721 PMC7233289 32096564

[4] Udugama B, Kadhiresan P, Kozlowski HN, Malekjahani A, Osborne M, Li VYC, Chen H, Mubareka S, Gubbay JB (2020). Chan WCW Diagnosing COVID-19: The disease and tools for detection. ACS Nano.

[5] Rajapaksha P, Elbourne A, Gangadoo S, Brown R, Cozzolino D, Chapman J (2019). A review of methods for the detection of pathogenic microorganisms. Analyst.

[6] Kelly-Cirino CD, Nkengasong J, Kettler H, Tongio I, Gay-Andrieu F, Escadafal C, Piot P, Peeling RW, Gadde R, Boehme C (2019). Importance of diagnostics in epidemic and pandemic preparedness. BMJ Glob. Health..

[7] Grubaugh ND, Ladner JT, Lemey P, Pybus OG, Rambaut A, Holmes EC, Andersen KG (2019). Tracking virus outbreaks in the twenty-first century. Nat. Microbiol..

[8] Bustin S, Huggett J (2017). qPCR primer design revisited. Biomol. Detect. Quantif..

[9] FDA [Jan 2021]. https://www.fda.gov/medical-devices/letters-health-care-providers/genetic-variants-sars-cov-2-may-lead-false-negative-results-molecular-tests-detection-sars-cov-2#:~:text=Health%20Care%20Providers-,Genetic%20Variants%20of%20SARS%2DCoV%2D2%20May%20Lead%20to%20False,Staff%20and%20Health%20Care%20Providers&text=The%20SARS%2DCoV%2D2%20virus,population%20of%20circulating%20viral%20strains

[10] Gu J, Han B, Wang J (2020). COVID-19: Gastrointestinal manifestations and potential fecal-oral transmission. Gastroenterology.

[11] Pan L (2020). Clinical characteristics of COVID19 patients with digestive symptoms in Hubei, China: A descriptive, cross-sectional, multicenter study. Am. J. Gastroenterol..

[12] Zhang, X. *et al*. The evidence of SARS-CoV-2 infection on ocular surface. *Ocul Surf*. 10.1016/j.jtos.2020.03.010 (2020). 10.1016/j.jtos.2020.03.010 PMC7194535 32289466

[13] Guo D, Xia J, Shen Y, Tong J (2020). SARS-CoV-2 may be related to conjunctivitis but not necessarily spread through the conjunctiva SARS-CoV-2 and conjunctiva. J. Med. Virol..

[14] Chen L, Liu M, Zhang Z, Qiao K, Huang T, Chen M, Xin N, Huang Z, Liu L, Zhang G, Wang J (2020). Ocular manifestations of a hospitalised patient with confirmed 2019 novel coronavirus disease. Br. J. Ophthalmol..

[15] Shu Y, McCauley J (2017). GISAID: Global initiative on sharing all influenza data: From vision to reality. Euro Surveill..

[16] Hatcher EL, Zhdanov SA, Bao Y, Blinkova O, Nawrocki EP, Ostapchuck Y, Schäffer AA, Brister JR (2017). Virus variation resource: Improved response to emergent viral outbreaks. Nucleic Acids Res..

[17] Sievers F, Higgins DG (2018). Clustal Omega for making accurate alignments of many protein sciences. Protein Sci..

[18] Capella-Gutiérrez S, Silla-Martínez JM, Gabaldón T (2009). trimAl: A tool for automated alignment trimming in large-scale phylogenetic analyses. Bioinformatics.

[19] Wu F, Zhao S, Yu B (2020). A new coronavirus associated with human respiratory disease in China. Nature.

[20] Ye J, Coulouris G, Zaretskaya I, Cutcutache I, Rozen S, Madden TL (2012). Primer-BLAST: A tool to design target-specific primers for polymerase chain reaction. BMC Bioinform..

[21] Rice P, Longden I, Bleasby A (2000). EMBOSS: The European molecular biology open software suite. Trends Genet..

[22] Camacho C, Coulouris G, Avagyan V, Ma N, Papadopoulos J, Bealer K, Madden TL (2009). BLAST+: Architecture and applications. BMC Bioinform..

