# Supplementary material for: Design and in silico validation of polymerase chain reaction primers to detect severe acute respiratory syndrome coronavirus 2 (SARS-CoV-2)
Source: Sci Rep. 2021 Jun 15;11:12565. doi: 10.1038/s41598-021-91817-9 (PMC8206341; doi:10.1038/s41598-021-91817-9)
Supplement: Supplementary file 1 — Supplementary Information 1. [file 41598_2021_91817_MOESM1_ESM.docx]

Supplementary table 1: number of hits with 0 mismatches based on the results from the probes similarity searches using standalone BLAST+ .

| **Probes** | **Hits with 0 mismatches** | **Total number of sequences** |
| --- | --- | --- |
| UFRN_P_1 | 199958 | 211833 |
| UFRN_P_2 | 199866 | 211833 |
| UFRN_P_3 | 200000 | 211833 |
| UFRN_P_4 | 199996 | 211833 |
| UFRN_P_5 | 200000 | 211833 |
| UFRN_P_6 | 200000 | 211833 |
| UFRN_P_7 | 200000 | 211833 |
| UFRN_P_8 | 200001 | 211833 |
| UFRN_P_9 | 200001 | 211833 |
