# Supplementary material for: Design and in silico validation of polymerase chain reaction primers to detect severe acute respiratory syndrome coronavirus 2 (SARS-CoV-2)
Source: Sci Rep. 2021 Jun 15;11:12565. doi: 10.1038/s41598-021-91817-9 (PMC8206341; doi:10.1038/s41598-021-91817-9)
Supplement: Supplementary file 2 — Supplementary Information 2. [file 41598_2021_91817_MOESM2_ESM.docx]

Supplementary table 2: Analysis of probes released by WHO (PD_primers set) to detect SARS-CoV-2 using PCR.

| **Probes** | **Variant** | **Hits without mismatches** | **Total number of sequences** |
| --- | --- | --- | --- |
| **Germany (17 January 2020)** | | |  |
| RdRp_SARSr-P2 | B.1.1.7 | 1931 | 1931 |
|  | B.1351 | 495 | 495 |
|  | P.1 | 177 | 177 |
|  | B.1429+1427+1525 | 94 | 94 |
|  |  |  |  |
| E_Sarbeco_P1 | B.1.1.7 | 1931 | 1931 |
|  | B.1351 | 495 | 495 |
|  | P.1 | 177 | 177 |
|  | B.1429+1427+1525 | 94 | 94 |
|  |  |  |  |
| N_Sarbeco_P1 | B.1.1.7 | 1925 | 1931 |
|  | B.1351 | 495 | 495 |
|  | P.1 | 176 | 177 |
|  | B.1429+1427+1525 | 94 | 94 |
| **Hong Kong (23 January 2020)** | | |  |
| HKU-ORF1b-nsp14P | B.1.1.7 | 0 | 1931 |
|  | B.1351 | 0 | 495 |
|  | P.1 | 0 | 177 |
|  | B.1429+1427+1525 | 0 | 94 |
|  |  |  |  |
| HKU-NP | B.1.1.7 | 1931 | 1931 |
|  | B.1351 | 495 | 495 |
|  | P.1 | 177 | 177 |
|  | B.1429+1427+1525 | 93 | 94 |
| **China (24 January 2020)** | | |  |
| ORF1ab_P | B.1.1.7 | 1929 | 1931 |
|  | B.1351 | 492 | 495 |
|  | P.1 | 177 | 177 |
|  | B.1429+1427+1525 | 94 | 94 |
|  |  |  |  |
| N_P | B.1.1.7 | 1924 | 1931 |
|  | B.1351 | 495 | 495 |
|  | P.1 | 177 | 177 |
|  | B.1429+1427+1525 | 94 | 94 |
| **Japan (24 January 2020)** | | |  |
| NIID_2019-nCOV_N_P | B.1.1.7 | 1931 | 1931 |
|  | B.1351 | 494 | 495 |
|  | P.1 | 177 | 177 |
|  | B.1429+1427+1525 | 94 | 94 |
| **Thailand (23 January 2020)** | | |  |
| WH-NIC_N_P | B.1.1.7 | 1931 | 1931 |
|  | B.1351 | 489 | 495 |
|  | P.1 | 177 | 177 |
|  | B.1429+1427+1525 | 94 | 94 |
|  |  |  |  |
| **USA (24 January 2020)** | | |  |
| 2019-nCoV_N1_P | B.1.1.7 | 1924 | 1931 |
|  | B.1351 | 486 | 495 |
|  | P.1 | 177 | 177 |
|  | B.1429+1427+1525 | 91 | 94 |
|  |  |  |  |
| 2019-nCoV_N2_P | B.1.1.7 | 1926 | 1931 |
|  | B.1351 | 494 | 495 |
|  | P.1 | 177 | 177 |
|  | B.1429+1427+1525 | 93 | 94 |
|  |  |  |  |
| 2019-nCoV_N3_P | B.1.1.7 | 1931 | 1931 |
|  | B.1351 | 494 | 495 |
|  | P.1 | 176 | 177 |
|  | B.1429+1427+1525 | 94 | 94 |
|  |  |  |  |
| RP_P | B.1.1.7 | 0 | 1931 |
|  | B.1351 | 0 | 495 |
|  | P.1 | 0 | 177 |
|  | B.1429+1427+1525 | 0 | 94 |
| **Paris (2 March 2020)** | | |  |
| nCoV_IP2-12696bProbe(+) | B.1.1.7 | 1928 | 1931 |
|  | B.1351 | 495 | 495 |
|  | P.1 | 177 | 177 |
|  | B.1429+1427+1525 | 94 | 94 |
|  |  |  |  |
| nCoV_IP4-14084Probe(+) | B.1.1.7 | 1929 | 1931 |
|  | B.1351 | 494 | 495 |
|  | P.1 | 177 | 177 |
|  | B.1429+1427+1525 | 94 | 94 |
|  |  |  |  |
| E_Sarbeco_P1 | B.1.1.7 | 1931 | 1931 |
|  | B.1351 | 495 | 495 |
|  | P.1 | 177 | 177 |
|  | B.1429+1427+1525 | 94 | 94 |
